# Two new species of *Acanthocotyle* Monticelli, 1888 (Monogenea: Acanthocotylidae), parasites of two deep-sea skates (Elasmobranchii: Rajiformes) in the South-East Pacific

**DOI:** 10.1186/s13071-019-3756-5

**Published:** 2019-10-30

**Authors:** Luis A. Ñacari, Fabiola A. Sepúlveda, Ruben Escribano, Marcelo E. Oliva

**Affiliations:** 1 Programa Doctorado Ciencias Aplicadas, Universidad de Antofagasta, Antofagasta, Chile; 2 Instituto Milenio de Oceanografía, Universidad de Concepción, Concepción, Chile; 3 Instituto Ciencias Naturales Alexander von Humboldt, Universidad de Antofagasta, Antofagasta, Chile

**Keywords:** Monogenea, *Acanthocotyle imo* n. sp., *Acanthocotyle atacamensis* n. sp., *Amblyraja frerichsi*, *Bathyraja peruana*, South-East Pacific Ocean, Deep-sea parasites, Skates

## Abstract

**Background:**

Parasites of deep-sea fishes from the South-East Pacific (SPO) are poorly known. Of *c.*1030 species of fish found in this area, 100–150 inhabit the deep-sea (deeper than 200 m). Only six articles concerning metazoan parasites of fish from deep-waters of SOP are known, and nine monogenean species have been reported. Currently, ten species are known in *Acanthocotyle* Monticelli, 1888 (Monogenea) and when stated, all of them are found in shallow waters (10–100 m). *Acanthocotyle gurgesiella* Ñacari, Sepulveda, Escribano & Oliva, 2018 is the only known species parasitizing deep-sea skates (350–450 m) in the SPO. The aim of this study was the description of two new species of *Acanthocotyle* from two Rajiformes.

**Methods:**

In September 2017, we examined specimens of two species of deep-sea skates (Rajiformes), *Amblyraja frerichsi* (Krefft) and *Bathyraja peruana* McEachran & Myyake, caught at *c.*1500 m depth off Tocopilla, northern Chile, as a by-catch of the Patagonian tooth fish *Dissostichus eleginoides* Smitt fishery. Specimens of *Acanthocotyle* were collected from the skin of the skates. Morphometric (including multivariate analysis of proportional measurements, standardized by total length), morphological and molecular analyses (*LSU* rRNA and *cox*1 genes) were performed in order to identify the collected specimens.

**Results:**

The three approaches used in this study strongly suggest the presence of two new species in the genus *Acanthocotyle*: *Acanthocotyle imo* n. sp. and *Acanthocotyle atacamensis* n. sp. parasitizing the skin of the thickbody skate *Amblyraja frerichsi* and the Peruvian skate *Bathyraja peruana*, respectively. The main morphological differences from the closely related species *Acanthocotyle verrilli* Goto, 1899 include the number of radial rows of sclerites, the non-discrete vitelline follicles and the number of testes.

**Conclusions:**

The two species of monogeneans described here are the only recorded parasites from their respective host species in the SPO. Assessing host specificity for members of *Acanthocotyle* requires clarifying the systematics of Rajiformes.
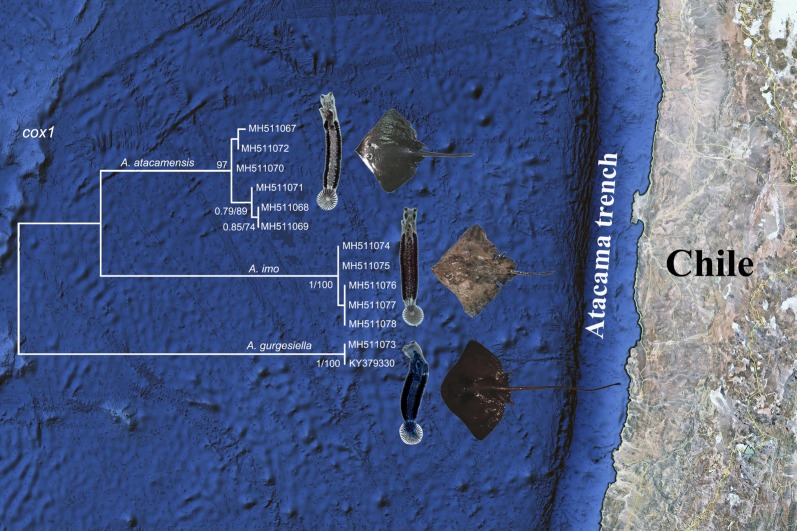

## Background

The deep-sea is one of the most fascinating ecosystems on earth [1], covering more than two-thirds of the world’s surface with an average depth of 3800 m and a maximum depth of *c.*11,000 m in Mariana Trench [2], but knowledge of biodiversity in this environment is still scarce [3]. Knowledge of biodiversity in the Atacama Trench, closely associated to the high productive Humboldt Current Marine Ecosystem is limited; the assemblage of deep-sea nematodes, the community of soft-shelled benthic foraminiferans and the presence of some amphipods have been described [4–6]. Surprisingly, the Atacama Trench is characterized by very high concentrations of nutritionally-rich organic matter up to depths of 7800 m, displaying characteristics typical of eutrophic systems [4]. The near-total lack of research on the parasites of deep-sea fish in the Atacama Trench represents an important gap in our knowledge of the biodiversity and structure of deep-sea communities in this trench [7] because host-parasite interactions may shape components of ecological communities [8]. Studies of the diversity of parasites of deep-sea fishes in the South-East Pacific (SPO), particularly for monogeneans are limited, and to date 11 monogenean species were recorded from deep-sea fishes in the SPO [7, 9, 10].

*Acanthocotyle* Monticelli, 1888 (Monopisthocotylea: Acanthocotylidae) includes parasites of the skin of skates and stingrays [11], which are considered specific parasites of Rajiformes, except for *Acanthocotyle urolophi* Kearn, Whittington, Chisholm & Evans-Gowing 2016, found in *Urolophus cruciatus* Lacépède (Myliobatiformes) and *Acanthocotyle* sp. from *Narcine maculata* (Shaw) (Torpediniformes) [12]. Ten valid species have been described to date: *Acanthocotyle lobianchi* Monticelli, 1888 (type-species); *A. elegans* Monticelli, 1890; *A. verrilli* Goto, 1899; *A. pacifica* Bonham & Guberlet, 1938; *A. pugetensis* Bonham & Guberlet, 1938; *A. williamsi* Price, 1938; *A. patagonica* Kuznetsova, 1971; *A. greeni* Macdonald & Llewellyn, 1980; *A. urolophi* Kearn, Whittington, Chisholm & Evans-Gowing 2016 and *A. gurgesiella* Ñacari, Sepulveda, Escribano & Oliva, 2018. Of these, only two species, *A. pacifica* and *A. gurgesiella* were described from deep-sea skates.

A detailed morphological and molecular study revealed that monogeneans obtained from the skin of two deep-sea skates *Amblyraja frerichsi* (Krefft) and *Bathyraja peruana* McEachran & Miyake, from SPO represent new species. These are described and differentiated below.

## Methods

### Sample collection and processing

In September 2017, ten specimens of both species, the thickbody skate *A. frerichsi* and Peruvian skate *B. peruana* (Rajiformes) were obtained as by-catch from the local demersal long-line fishery on Patagonian tootfish *Dissostichus eleginoides* Smitt (Nototheniidae) in SPO (off Tocopilla, northern Chile; 22°16′S, 70°38′W–23°26′S, 70°43′W) caught at depths of *c.*1500 m. The fish were immediately frozen (at − 18 °C) on board and transported to the laboratory for parasitological analysis. The dorsal surface was washed in tap water, and the mucus was sieved and examined for monogeneans using a dissecting microscope with transmitted light. Some monogeneans were fixed in AFA (alcohol: formalin: acetic acid; 1:1:8) or 4% neutral buffered formaldehyde and then transferred and stored in 70% ethanol for further morphological analyses (light microscopy). Selected monogeneans from each of the two hosts were transferred to 96% ethanol for DNA analyses.

Population descriptors, prevalence and mean intensity [13] were recorded for both parasite species.

### Morphological and statistical analyses

Fixed specimens were stained with Gomori’s trichrome and cleared with clove oil (Sigma-Aldrich, Taufkirchen, Germany), then mounted in Eukitts® (O. Kindler GmBH, Freiburg, Germany). The specimens were photographed (Leica M125 camera, Wetzlar, Germany) and measured using ImageJ [14]. Figures were made with a drawing tube. Measurements are in micrometers and are given as the range followed by the mean and the number of structures measured or counted in parentheses. The type-material was submitted to the National Museum of Natural History of the Smithsonian Institution, Washington, USA (NMNH-SI), Museo de Historia Natural, Universidad Nacional Mayor de San Marcos, Lima, Perú (MHN-UNMSM) and the Natural History Museum, London, UK (NHMUK).

To comply with the regulations set out in article 8.5 (amended version 2012) of the *International Code of Zoological Nomenclatures* (ICZN), details of the paper have been submitted to ZooBank. The LSID (Life Science Identifier) is urn:lsid:zoobank.org:pub:61DF198B-CF21-4B5E-B9BF-D7B80B187E49.

Principal components analysis (PCA) was performed for proportional morphometric measurements [15]. The ratios body width/total length (TL), body length (excluding posthaptor)/TL, pharynx length/TL, pharynx width/TL, diameter of the pseudohaptor/TL, number of sclerite rows/TL, testes maximum width/TL, germarium length/TL and germarium width/TL were used instead of the original measurements because previous results indicated a correlation between the morphological variables and the total length [16]. Subsequently, the first five main components of the PCA, explaining 90.4% of the variance, were used in a multivariate discriminant analysis (MDA). Statistical analyses were performed with Statistica 10.0.

### Molecular data and phylogenetic analyses

Parasites were preserved in 95% ethanol and placed individually into 1.5 ml Eppendorf tubes for DNA extraction. The DNA of each individual was isolated following a modified protocol [17], involving treatment with sodium dodecyl sulfate, digestion with Proteinase K, NaCl protein precipitation, and subsequent ethanol precipitation of the DNA.

For molecular analyses, regions within the nuclear *LSU* rRNA and the mitochondrial gene cytochrome *c* oxidase 1 (*cox*1) were used. *LSU* rRNA was amplified by polymerase chain reaction (PCR) with the forward primer C1 (5′-ACC CGC TGA ATT TAA GCA T-3′) and the reverse primer D2 (5′-TGG TCC GTG TTT CAA GAC-3′) [18]; *cox*1 was amplified using the forward primer L-CO1 (5′-TTT TTT GGG CAT CCT GAG GTT TAT-3′) and the reverse primer H-COX1 (5′-TAA AGA AAG AAC ATA ATG AAA ATG-3′) [19].

Each PCR reaction had a final volume of 35 μl including: 5 standard units of GoTaq DNA polymerase (Promega, Madison, USA), 7 μl 5× PCR buffer, 5.6 μl MgCl_2_ (25 mM), 2.1 μl BSA (10 mg/ml), 0.7 μl of deoxynucleotide triphosphate (dNTP) (10 mM), 10 pM of each primer and 7 μl template DNA. A Boeco Ecogermany M-240R Thermal Cycler (Boeckel, Hamburg, Germany) was used with a cycling profile as follows: 30 temperature cycles programmed on a slow temperature ramp rate. Cycle 1 was 95 °C for 3 min, 45 °C for 2 min and 72 °C for 90 s. This was followed by four cycles of 95 °C for 45 s, 50 °C for 45 s and 72 °C for 90 s, then a further 25 cycles of 95 °C for 20 s, 52 °C for 20 s and 72 °C for 90 s. The mix was held at 72 °C for 5 min to complete extension and then dropped to 4 °C. For *cox*1 PCR, there was an initial denaturation step at 95 °C (5 min) followed by 35 cycles of 95 °C for 1 min, 48 °C for 2 min and 72 °C for 21 min) with a final extension step at 72 °C for 10 min. PCR products were directly sequenced (Macrogen, Seoul, Korea; http://www.macrogen.com).

Sequences were edited and assembled using ProSeq v2.9 [20]. The fragments obtained from the *LSU* rRNA gene were aligned using the Clustal 2 [21] software package with sequences of related monogeneans retrieved from GenBank (Table 1). All new DNA sequences were deposited in the GenBank database, and the accession numbers are given in Table 1.

**Table 1:** GenBank accession numbers for sequences (*LSU* rRNA gene and *cox*1 gene) for *Acanthocotyle* spp. and the species of the outgroup used in phylogenetic analyses

Species	Host	Locality	GenBank ID		References
			*LSU* rRNA	*cox*1	
*A. atacamensis* n. sp.	*Bathyraja peruana* McEachran & Myyake	Off Tocopilla, northern Chile (22°16′S, 70°38′W–23°26′S, 70°43′W)	MH511079–MH511082	MH511067–MH511072	This study
*A. gurgesiella* Ñacari, Sepúlveda, Escribano & Oliva, 2018	*Gurgesiella furvescens* de Buen	Off Valparaiso, central Chile (33°S, 72°W)	KY379328–KY379329	KY379330–KY379331	[10]
*A. imo* n. sp.	*Amblyraja frerichsi* (Krefft)	Off Tocopilla, northern Chile (22°16′S, 70°38′W–23°26′S, 70°43′W)	MH511083–MH511085	MH511074–MH511078	This study
*A. urolophi* Kearn, Whittington, Chisholm & Evans-Gowing, 2016	*Urolophus cruciatus* (Lacépède)	Tasman Sea (off Australia)	FJ971962		[11]
Outgroup					
Amphibdellatidae gen. sp.	*Narcine tasmaniensi* (Richardson)	Off Australia	FJ971964		[23]
*Neocalceostoma* sp.	*Arius venosus* Valenciennes	Off Malaysia	AF387510		[24]

Phylogenetic reconstruction was performed using Bayesian inference (BI) and maximum-likelihood (ML) analyses. jModelTest 0.1.1 software [22] was employed to determine the best-fit nucleotide substitution model under the Akaike information criterion AIC [23]. For *LSU* rRNA and *cox*1 genes, the models GTR + G and GTR + I, respectively, were used as optimal models. For BI, unique random starting trees were used in the Metropolis-coupled Markov chain Monte Carlo (MCMC [24]. For both genes, independent MCMC chains were run with 50,000,000 of generations, sampling every 1000 generations, obtaining effective samples sizes (ESS) of parameter estimates over 200. Results were visualized in TRACER v. 1.7 [25]. ML analysis was performed using the MEGA v.6 considering gaps [26], missing data, pairwise deletions, codon positions, and 1st + 2nd + 3rd + non-coding positions. Nodal support was estimated by bootstrapping (*n* = 1000).

The sequences of the monogeneans Amphibdellatidae gen. sp. (GenBank: FJ971964) and *Neocalceostoma* sp. (GenBank: AF387510) were used as the outgroup for *LSU* rRNA phylogenetic tree [27, 28]. No sequences for *cox*1 gene are available on GenBank for potential outgroups in the phylogenetic tree. Pairwise p-distances were also calculated with MEGA v6.

## Results

**Class Monogenea (van Beneden, 1858)**

**Family Acanthocotylidae Monticelli, 1903**

**Genus** ***Acanthocotyle*** **Monticelli, 1888**

***Acanthocotyle imo*** **n. sp.**

***Type-host***: *Amblyraja frerichsi* (Krefft) (Rajiformes: Rajidae), thickbody skate.

***Type-locality***: Off Tocopilla, northern Chile (22°16′S, 70°38′W–23°26′S, 70°43′W) at *c*.1500 m depth.

***Type-material***: The holotype (NMHH-SI 1480277) and 2 paratypes (NMNH-SI 1480278-9) were submitted to the National Museum of Natural History, Smithsonian Institution, Washington D.C., USA; 2 paratypes (MHN-UNMSM 3645-3646) were submitted to the Museo de Historia Natural, Universidad Nacional Mayor de San Marcos, Lima, Peru; and 2 paratypes (NHMUK 2018.8.30.1-2,) were submitted to the Natural History Museum, London, UK.

***Site on host***: Skin.

***Prevalence and intensity***: Prevalence: 100%; mean intensity: 2.8.

***Representative DNA sequences***: MH511083-MH511085 (*LSU* rRNA gene); MH511074-MH511078 (*cox*1 gene).

***Etymology***: The specific name refers to the Instituto Milenio de Oceanografia (IMO), Chile, in appreciation of the financial and logistic support.

### Description

**Fig. 1:**
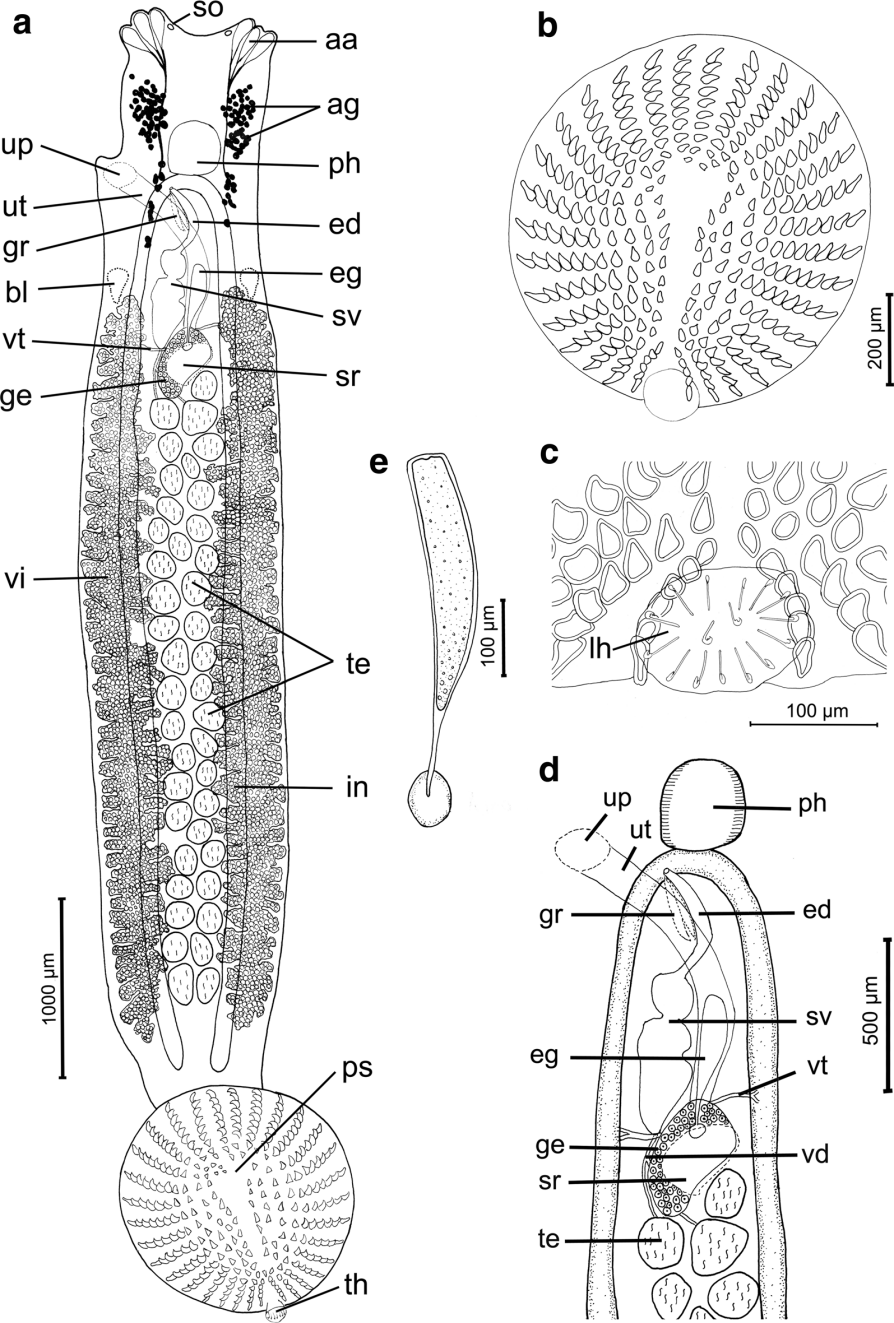
*Acanthocotyle imo* n. sp. ex *Amblyraja frerichsi.* **a** Ventral view of the holotype. **b** Pseudohaptor. **c** Details of true haptor. **d** Reproductive system. **e** Egg. *Abbreviations*: aa, anterior adhesive lobes; ag, accessory glands; bl, excretory bladders; ed, ejaculatory duct; eg, egg; ge, germarium; gr, male accessory gland reservoir; in, intestine; lh, larval hooks; ph, pharynx; ps, pseudohaptor; so, sense organs; sr, seminal receptacle; sv, bipartite seminal vesicle; te, testes; th, true haptor; up, uterine atrium; ut, uterus; vd, vas deferens; vt, vitelline duct; vi, vitelline follicles

[Measurements based on 21 adult worms. Metrical data and meristic counts were taken for each parasite (Table 2). Raw measurements are given in Additional file 1: Table S1.] Total body length, including pseudohaptor 3418–8434 (5600); maximum body width 512–1440 (867) (Fig. 1a). True haptor with 16 marginal hooks (14 peripheral and 2 central) (Fig. 1c) located at posterior margin of pseudohaptor. Pseudohaptor nearly circular, 704–1473 (1086) in length and 667–1466 (1038) in width, with 30–35 (mode 32) rows of sclerites with sharp recurved tips (Fig. 1b); rows are easily counted peripherally; each row consists of 6–10 sclerites [total number 268–314 (292)] (Fig. 1b). Marginal valve of pseudohaptor smooth. Pharynx globular, 163–354 × 132–388 (248 × 231) (*n* = 20). Three anterior adhesive lobes present on each side of anterior extremity, with a single aperture. Sensory organs conspicuous, at or near anterior margin of head. Excretory bladders 2, anterior to vitellarium field on each side of body. More or less pronounced constriction (or neck) present at level of posterior margin of pharynx, marking off head region from body proper. Intestinal caeca without diverticula, not confluent. Eyes absent. Testes mainly rounded, 32–47 (mode 41) in number, posterior to germarium, usually arranged in 2 rows, 92–234 × 80 − 213 (151 × 138). Vas deferens connecting posterior seminal vesicle slightly lobed; anterior seminal vesicle smooth, communicates with male genital opening *via* relatively large, curved ejaculatory duct; male accessory gland reservoirs 2, adjacent to ejaculatory duct (Fig. 1d). Penis unarmed; male genital opening unarmed, slightly dextral, at level of intestinal bifurcation. Germarium subglobular 104–412 (236, *n* = 17) long, 149–352 (219, *n* = 19) wide. Uterine seminal receptacle 104–294 × 98–246 (199 × 173) (*n* = 16), contiguous with germarium; vagina absent. Germinal appendix not observed. Opening of uterine atrium dextral and dorsal, at level of posterior part of pharynx (Fig. 1d). Eggs sausage-shaped, 220–380 × 60–110 (290 × 80) (*n* = 11), with abopercular appendages, 90–210 (120, *n* = 11). Eggs (Fig. 1e) observed in 11 specimens, but each one harbored only one egg. Vitellarium extracaecal, consisting of numerous elongated, non-discrete follicles, extending from level of posterior seminal vesicle to near posterior end of body proper.

**Table 2:** Meristic and morphometric data for *Acanthocotyle* spp. from off northern Chile

Species	*A. imo* n. sp.	*A. atacamensis* n. sp.	*A. gurgesiella* Ñacari, Sepúlveda, Escribano & Oliva, 2018
Host	*Amblyraja frerichsi*	*Bathyraja peruana*	*Gurgesiella furvescens*
Source	Present study (*n* = 21) Range (mean)	Present study (*n* = 21) Range (mean)	Ñacari et al. [10] (*n* = 10) Range (mean)
Body length	3418–8434 (5600)	2571–7702 (5087)	3070–5720 (4190)
Maximum body width	512–1440 (867)	292–931 (689)	630–1180 (940)
Pharynx length	163–354 (248)	186–346 (262)	110–330 (240)
Pharynx width	132–388 (231)	152–309 (234)	150–360 (250)
Pseudohaptor length	704–1473 (1086)	487–1085 (862)	600–1100 (890)
Pseudohaptor width	667–1466 (1038)	416–1075 (840)	640–1250 (910)
No. of sclerite rows in pseudohaptor	30–35 (mode 32)	28	36–40 (mode 38)
Total no. of sclerites in pseudohaptor	268–314 (292)	186–240 (220)	201–331 (270)
No. of testes	32–47 (mode 41)	40–58 (mode 50)	28–43 (mode 35)
Maximum testis width	80–213 (138)	186–346 (262)	30–80 (70)
Germarium length	104–412 (236)	75–243 (159)	130–280 (240)
Germarium width	149–352 (219)	141–267 (204)	160–290 (240)
Seminal receptacle length	104–294 (199)	111–269 (193)	63
Seminal receptacle width	98–246 (173)	103–216 (150)	54–62 (59)
Egg length	218–381 (287)	305–336 (321)	–
Egg width	56–113 (76)	64–93 (79)	–

### Differential diagnosis

**Fig. 2:**
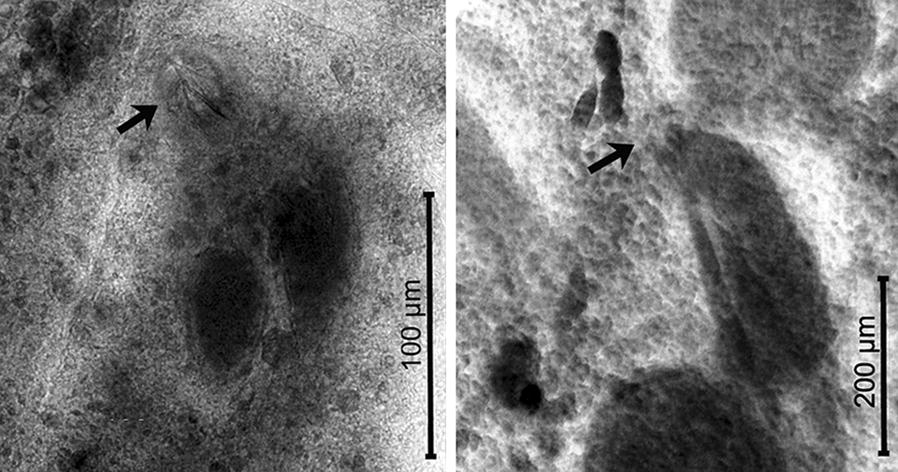
Photomicrography of penis armed with spear-shaped spine in *Acanthocotyle gurgesiella* (left, arrow) and unarmed penis in *A. imo* (right, arrow)

*Acanthocotyle imo* n. sp. is morphologically similar to *A. verrilli* and *A. gurgesiella*, species with a pseudohaptor armed with 21–39 radial rows of sclerites, having a dextral opening of uterine atrium, non-discrete vitelline follicles, and more than 20 testes [11]. The number of radial rows of sclerites has been reported to range from 30 to 34 in *A. verrilli* [11] and between 36–40 (mode 40) in *A. gurgesiella* [*vs* 30–35 (mode 32)] in the new species. The number of testes in the new species (32–47; mode 41) agree well with values given for *A. verrilli* (range from 37 to *c.*57) [29, 31] and *A. gurgesiella* (28–43) [10], but the mode (30) for the latter species is lower than for *A. imo* n. sp. The testes in *A. verrilli* are arranged in numerous rows (*vs* mainly in two rows in *A. imo* n. sp. and *A. gurgesiella*). The ratio total length/pseudohaptor is higher in the new species (4.61–5.86) compared with *A. verrilli* (2.48–2.86) [29, 31], but similar to the ratio in *A. gurgesiella* (4.37–5.10) [10]. The presence of a smooth marginal valve of the pseudohaptor in *A. imo* n. sp. instead of a marginal valve with a distinct fringe in *A. verrilli* is an additional difference between the two species. *Acanthocotyle imo* n. sp. can be readily differentiated from *A*. *gurgesiella* by the lack of a spear-shaped spine in penis (present in the latter species) (see Fig. 2 and Additional file 2: Table S2).

***Acanthocotyle atacamensis*** **n. sp.**

***Type-host***: *Bathyraja peruana* McEachran & Miyake, 1984 (Rajiformes: Arhynchobatidae), Peruvian skate.

***Type-locality***: Off Tocopilla, northern Chile (22°16′S, 70°38′W–23°26′S, 70°43′W), at *c*.1500 m depth.

***Type-material***: The holotype (NMNH-SI 1480280) and 2 paratypes (NMNH-SI 1480281-2) were submitted to the National Museum of Natural History, Smithsonian Institution, Washington D.C., USA; 2 paratypes (MHN-UNMSM 3643-3644) were submitted to the Museo de Historia Natural, Universidad Nacional Mayor de San Marcos, Lima, Peru; and 2 paratypes (NHMUK 2018.8.30.3-4,) were submitted to the Natural History Museum, London, UK.

***Site on host***: Skin.

***Prevalence and intensity***: Prevalence: 100%; Mean intensity: 3.0.

***Representative DNA sequences***: MH511079-MH511082 (*LSU* rRNA gene); MH511067-MH511072 (*cox*1 gene).

***Etymology:*** The specific name of the new species refers to the Atacama trench where samples were obtained.

### Description

**Fig. 3:**
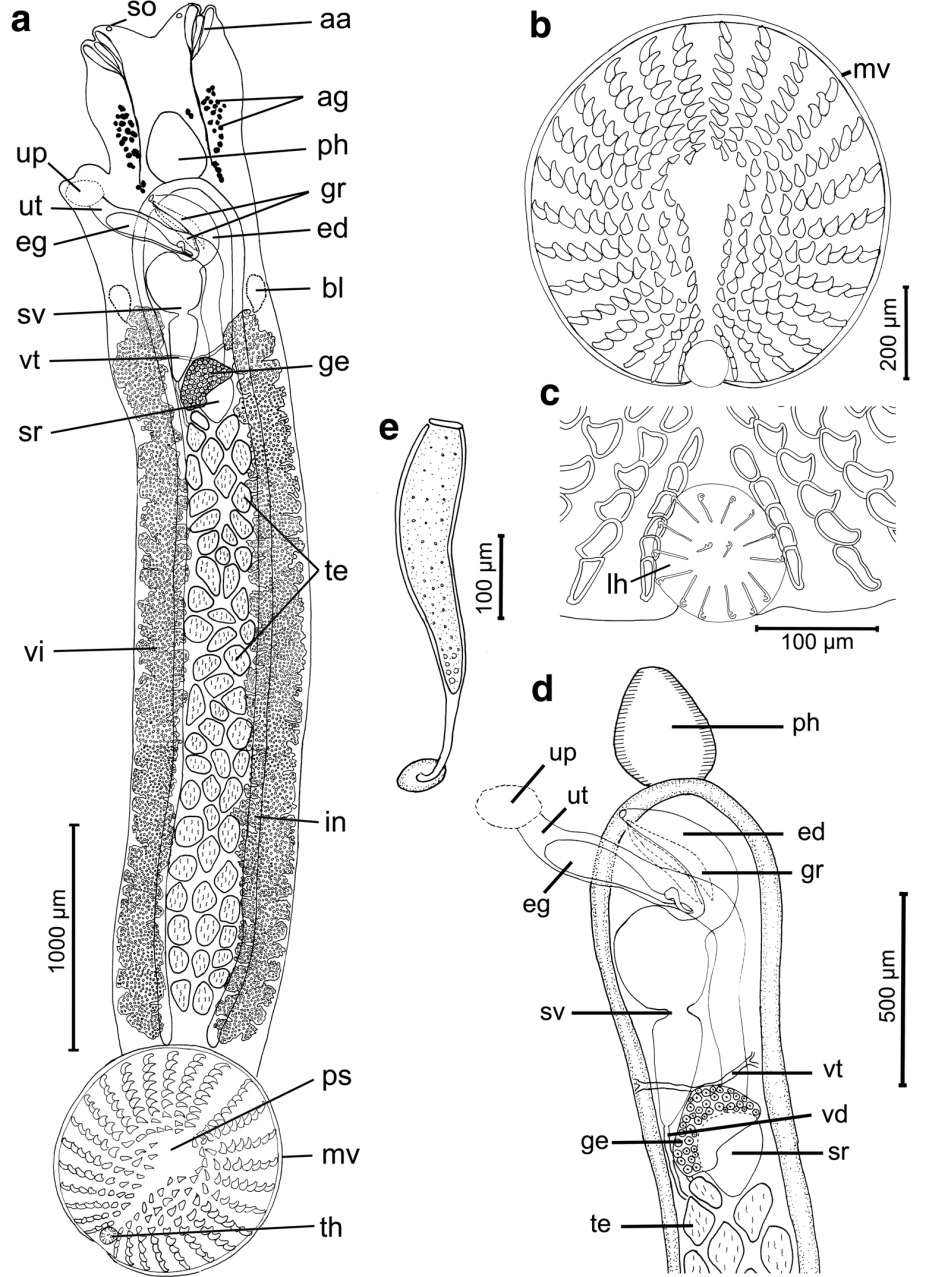
*Acanthocotyle atacamensis* n. sp. ex *Bathyraja peruana*. **a** Ventral view of the holotype. **b** Pseudohaptor. **c** Details of true haptor. **d** Reproductive system. **e** Egg. *Abbreviations*: aa, anterior adhesive lobes; ag, accessory glands; bl, excretory bladders; ed, ejaculatory duct; eg, egg; ge, germarium; gr, male accessory gland reservoir; in, intestine; lh, larval hooks; mv, marginal valve; ph, pharynx; ps, pseudohaptor; so, sense organs; sr, seminal receptacle; sv, bipartite seminal vesicle; te, testes; th, true haptor; up, uterine atrium; ut, uterus; vd; vas deferens; vd vitelline duct; vi, vitelline follicles

**Fig. 4:**
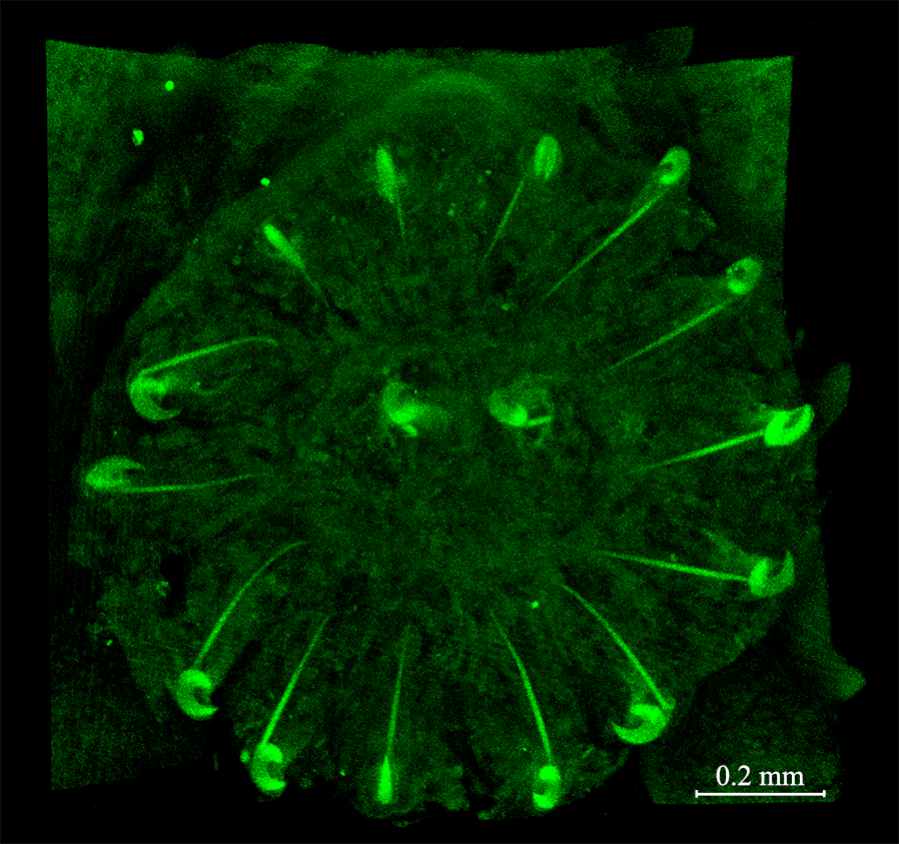
Confocal image of the pseudohaptor of *A. atacamensis* n. sp. ex *Bathyraja peruana*

[Measurements based on 21 adult worms. Metrical data and meristic counts were taken for each parasite (Table 2). Raw measurements are given in Additional file 3: Table S3.] Total body length, including pseudohaptor 2571–7702 (5087); maximum body width 292–931 (689) (Fig. 3a). True haptor with 16 marginal hooks (14 peripheral and 2 central) (Figs. 3c, 4) located at posterior margin of pseudohaptor (Fig. 3b). Pseudohaptor nearly circular, 487–1085 (862) in length by 416–1075 (840) in width, with 28 rows of sclerites with sharp recurved tips (Fig. 3b); rows are easily counted peripherally; each row consists of 6–10 sclerites [total number 186–240 (220)] (Fig. 3b). Marginal valve of pseudohaptor with distinct fringe (Fig. 3b). Pharynx globular, 185–346 × 152–309 (262 × 234) (*n* = 20). Three anterior adhesive lobes present on each side of anterior extremity, with a single aperture. Sensory organs conspicuous at or near anterior margin of head. Excretory bladders 2, anterior to vitellarium field on each side of the body. More or less pronounced constriction (or neck) present at level of posterior margin of pharynx, marking off head region from body proper. Intestinal caeca without diverticula. Eyes absent. Testes irregular in shape, 40–58 (mode 50) in number, posterior to germarium, usually arranged in 3 rows, 70–178 × 41–131 (122 × 92). Vas deferens expands conforming unlobed posterior seminal vesicle and anterior unlobed seminal vesicle that communicates with male genital opening *via* relatively large, curved ejaculatory duct; male accessory gland reservoirs 2, adjacent to ejaculatory duct (Fig. 3d). Penis unarmed; male genital opening unarmed, slightly dextral, at level of intestinal bifurcation. Germarium subglobular, 75–243 (149, *n* = 19) long, 141–267 (204) (*n* = 19). Uterine seminal receptacle 111–279 × 103–216 (193 × 150) (*n* = 16), contiguous with germarium; vagina absent. Germinal appendix not observed. Opening of uterine atrium dextral and dorsal, at level of posterior part of pharynx (Fig. 3d). Eggs sausage-shaped, 305–336 × 64–93 (321 × 79) (*n* = 2) (Fig. 3e) with abopercular appendages 111–116 (114) (*n* = 2). Eggs observed in 2 specimens, but each one harbored only one egg. Vitellarium extracaecal, consisting of numerous elongate follicles, extending from the level of the posterior external seminal vesicle to near the posterior end of body proper.

### Differential diagnosis

*Acanthocotyle atacamensis* n. sp. resembles morphologically *A. verrilli* and *A. imo* n. sp. characterized by possessing more than 10 testes, a pseudohaptor armed with 21–40 radial rows of sclerites, a dextral opening uterine atrium and non-discrete vitelline follicles [11]. The number of the testes overlaps in the three species: 37–*c.*57 in *A. verrilli* [29, 30]; 32–47 (mode 41) in *A. imo* n. sp.; and 40–58 (mode 50) in *A. atacamensis* n. sp. The main differences with *A. verrilli* and *A. imo* n. sp. include the number of radial rows of sclerites ranging between 30–34 in *A. verrilli*, 30–35 in *A. imo* n. sp. instead of 28 (no variation) in *A. atacamensis*. Similarly, the total number of sclerites is greater in *A. verrilli* (387) and *A imo* (268–314) than in *A. atacamensis* n. sp. (186–240). Additionally, the ratio TL/pseudohaptor is greater in *A. atacamensis* n. sp. (5.03–7.80) and *A. imo* n. sp. (4.61–5.86) than in *A. verrilli* (2.76–2.86), indicating a pseudohaptor that is proportionally larger in *A. verrilli.* A summary of the morphological and morphometric characteristics of the species of *Acanthocotyle* considered valid are provided in Additional file 2: Table S2.

Notably, the three species of *Acanthocotyle* from the SPO harbored a single egg and not egg bundles as indicated for other species in the genus.

### Morphometric analysis

**Fig. 5:**
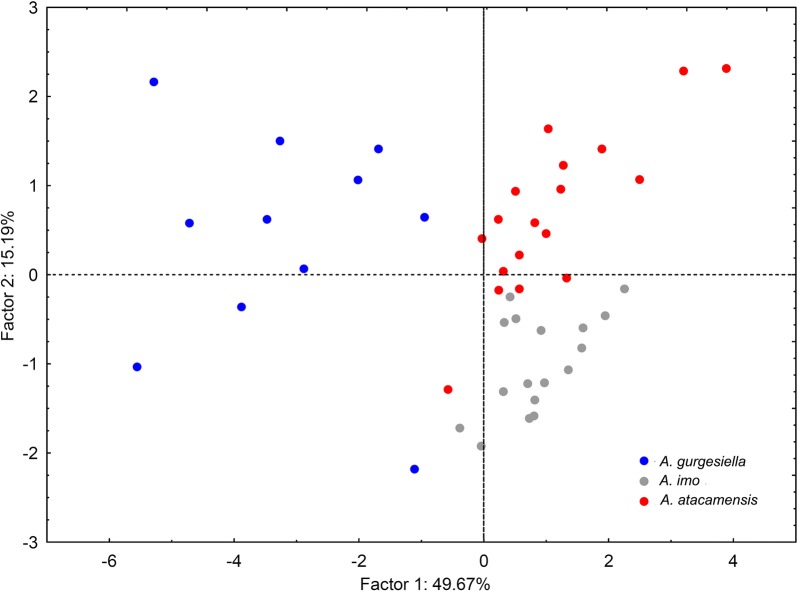
Principal components analysis based on proportional morphometric measurements standardized by total length from three species of *Acanthocotyle* from off northern Chile. Blue circles: *A. gurgesiella* ex *Gurgesiella furvescens*; grey circles: *A. imo* n. sp. ex *Amblyraja frerichsi*; red circles: *A. atacamensis* n. sp. ex *Bathyraja peruana*

Figure 5 presents a plot of the specimens in the two-dimensional plane of the PCA. The first and second components of the PCA explained 64.86% of the total variance. The first component explaining 49.67% of the variance was associated with the proportional morphometric measurements of body width/TL, body length (excluding pseudohaptor)/TL, pharynx width/TL and germarium length/TL, whereas the second component explaining 15.19% of the variance was associated with pharynx length/TL, testes width/TL and germarium width/TL.

The results of the multivariate discriminant analysis (MDA) (Table 3) showed a correct assignment for the three species that, on average, reached 93.5% of the studied specimens of *Acanthocotyle* (Wilk’s lambda = 0.78, *F*_(10, 78)_ = 20.17, *P* < 0.001). The probability of correct assignment by chance alone was 36.2%.

**Table 3:** Classification matrix of the multivariate discriminant analysis, based on morphometrics proportion standardized by total length of three species of *Acanthocotyle* from the South-East Pacific Ocean

Species	*A. imo* (*P* = 0.37)	*A. atacamensis* (*P* = 0.41)	*A. gurgesiella* (*P* = 0.22)	% Correct classification
*A. imo*	16	1	0	94.1
*A. atacamensis*	1	18	0	94.7
*A. gurgesiella*	1	0	9	90.0
Total	18	19	9	93.5

*Note*: Pairwise probabilities all < 0.001

### Molecular and phylogenetic analyses

For the *LSU* rRNA region, seven sequences of 862 bp were obtained (4 from *A. atacamensis* n. sp. and 3 from *A. imo* n. sp.). Intraspecific genetic variability for both *A. imo* and *A. atacamensis* was 0%. Sequences were aligned and trimmed to 409 bp (the size of sequences available on GenBank) in order to compare the new species with *A. gurgesiella* and *A. urolophi*. Genetic distances between the new species and *A. gurgesiella* and *A. urolophi* are shown in Table 4.

**Table 4:** Percent pairwise genetic distances between *Acanthocotyle* spp. for *LSU* rRNA gene (under the diagonal) and the mitochondrial *cox*1 gene (above the diagonal)

	*A. atacamensis* (*n* = 6)	*A. imo* (*n* = 5)	*A. gurgesiella* (*n* = 2)
*A. atacamensis* (*n* = 4)	–	14.3–15.6	18.8–19.5
*A. imo* (*n* = 3)	0.50	–	21.5–21.8
*A. gurgesiella* (*n* = 2)	2.24–2.74	1.75–2.24	–
*A. urolophi* (*n* = 1)	2.99	2.74	3.24–3.74

*Notes*: Comparisons are based on 409 bp (*LSU* rRNA gene) and 307 bp (*cox*1). Numbers in parentheses indicate the number of sequences used for the comparisons (*LSU* rRNA gene, first column; *cox*1, first line)

Eleven sequences of the *cox*1 gene were obtained: 6 from *A. atacamensis* n. sp. (713 bp); 5 from *A. imo* n. sp. (669 bp); 2 published sequences from *A. gurgesiella* (424 bp) [10] were also included in analysis. Intraspecific variability ranged from between 0–0.7% (5 polymorphic sites) for *A. atacamensis* n. sp., 0–0.3% for *A. imo* n. sp. (3 polymorphic sites) and 0.5% for *A. gurgesiella* (2 polymorphic sites). Sequences were aligned and trimmed to 307 bp for comparison with *A. gurgesiella.* Genetic distances between the two new species and *A. gurgersiella* are shown in Table 4.

**Fig. 6:**
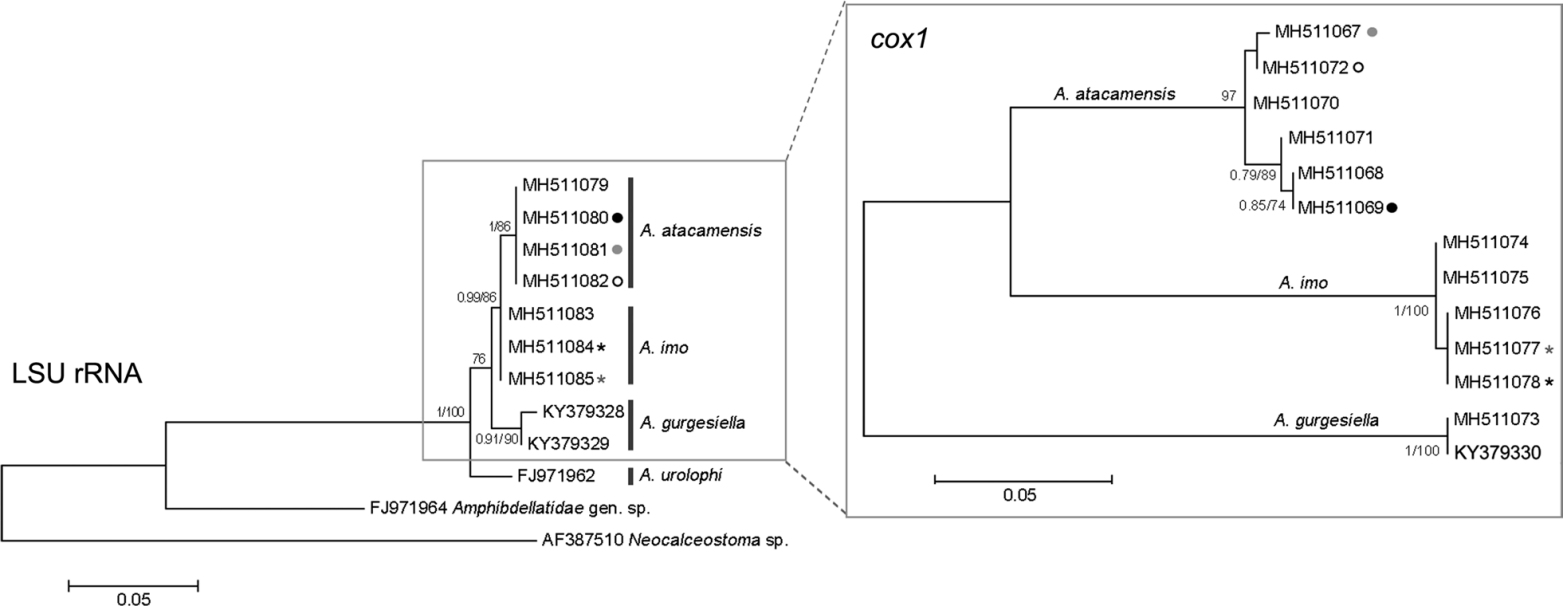
Phylogenetic trees based on *LSU* rRNA and *cox*1 genes for *Acanthocotyle* spp. from different rajiformes host species, inferred by Bayesian inference (BI) and Maximum Likelihood (ML). Numbers along branches indicate the bootstrap values obtained from the posterior probability of BI and ML analysis (BI/ML); only values > 0.95 (BI) and > 70 (ML) are shown. Sequences for Amphibdellatidae gen. sp. and *Neocalceostoma* sp. were used as the outgroup for *LSU* rRNA gene analysis

The trees in Fig. 6 show the phylogenetic relationships based on the *LSU* rRNA and *cox*1 genes for members of *Acanthocotyle*. *LSU* rRNA gene suggest that sequences from conspecific specimens of *Acanthocotyle* spp. clustered together in a single monophyletic clade, supported by high posterior probability (BI = 1) and high bootstrap support value from the ML analysis (ML = 100). *LSU* rRNA data did not support reciprocal monophyly of *A. imo* n. sp. but this was supported by the more variable mitochondrial gene, *cox*1. Unrooted *cox*1 tree in Fig. 6 shows the sequences of the two new species from South-East Pacific (*A. atacamensis* n. sp. and *A. imo* n. sp.) and *A. gurgesiella* forming three well supported clades.

## Discussion

Traditional taxonomy based on morphology and morphometry and multivariate analyses based on morphometric data corrected for body length, strongly supports three species of *Acanthocotyle* detected in three different skates (all members of Rajiformes) from SPO (off Tocopilla, northern Chile). *LSU* rRNA data did not support reciprocal monophyly for *A. imo* n. sp. but this was supported by the *cox*1 gene data. Absence of reciprocal monophyly could be a consequence of a short length of the studied fragment and/or the gene may not be variable enough to reflect recent divergence.

The lack of molecular data from almost all members of *Acanthocotyle* (except for *A. urolophi* and *A. gurgesiella*) precludes the molecular confirmation of the species described herein, but morphological characteristics of the new species are robust enough to confirm their distinct status. The two new species are easily differentiated from their congeners by a combination of characteristics that includes morphometric and morphological characters as indicated in the differential diagnosis for each species.

Including the two new species described above, *Acanthocotyle*, the unique genus in the Acanthocotylidae, now comprises 12 valid species. Only two species have been reported from hosts other than Rajiformes: *A. urolophi* described from *Urolophus cruciatus* (Myliobatiformes) [11] and *Acanthocotyle* sp. recorded from *Raja clavata* but also *Narcine maculata* (Torpediniformes); no differences between specimens obtained from the two hosts were indicated [12]. The presence of *Acanthocotyle* sp. in *N. maculata* could be the result of a transfer of the parasite between fishes during capture. A similar reason was suggested to explain the presence of *A. williamsi* (as *Pseudacanthocotyla williamsi*) in the teleost *Sebastes alutus* (Gilbert) (Scorpaeniformes) [32]. Surprisingly, this species was also recorded from the gills of another teleost fish, *Reinhardtius hippoglossoides* (Walbaum) (Pleuronectiformes) [33]. Unfortunately, we were unable to find additional records of *A. williamsi* in order to check the identity of the host. A different picture is evident for *A. verrilli*, also described originally for a “skate” but at least seven records, from four members of the Rajidae, are available. Species of *Acanthocotyle* seem to be specific to members of the Rajidae, but this assumption must be treated with caution. During 2016, three major contributions to the taxonomy of elasmobranchs were published [34–36]. Accordingly, hosts for *Acanthocotyle* are included not only in the Rajidae [32] but also in the Arhynchobatidae [34, 35]. The specificity of *Acanthocotyle*, at least at the family level, requires clarifying the systematics of the Rajiformes.

The integration of both, molecular and morphological tools and discriminant morphometric characters has strongly strengthened the traditional taxonomy, resolving the existence of cryptic species, identification of new species, and also clarification of species taxonomic status [16, 37–39]. Thus, our findings, based on molecular and morphometric multivariate analysis, are strongly consistent. The results of PCA and *cox*1 genes support the same conclusion: the species that are closer in the first plane of the PCA plot (*A. imo* n. sp. and *A. atacamensis* n. sp., see Fig. 5), also appear closer in the phylogenetic tree based on *cox*1 but reciprocal monophyly for *A. imo* is not supported by *LSU* rRNA (Fig. 6). These results clearly suggest the key importance of integrating molecular and multivariate morphometric analyses for taxonomic studies.

**Fig. 7:**
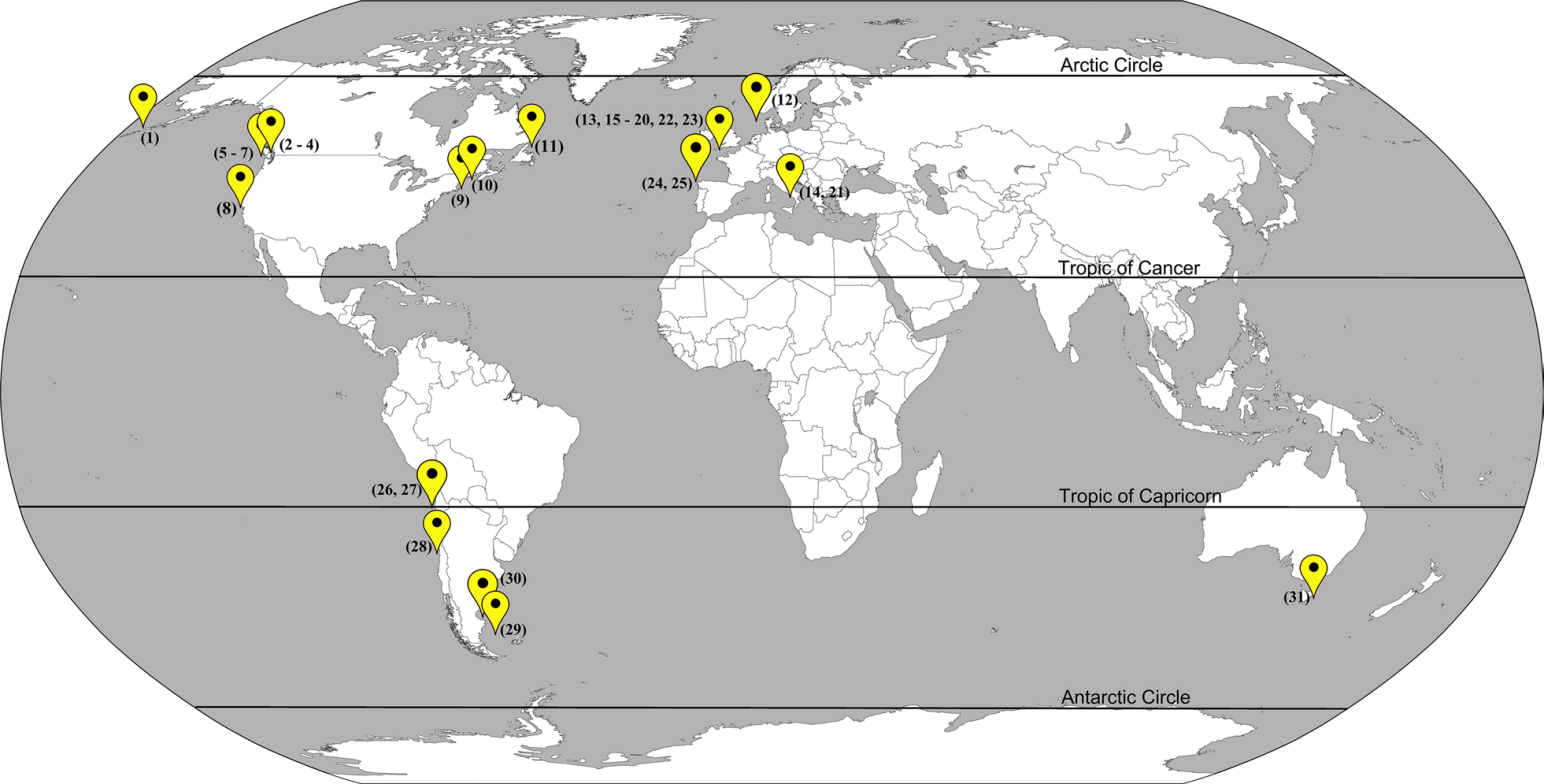
Distribution map of the known species of *Acanthocotyle. Key*: 1, *A. williamsi* ex unidentified skate, Bering Sea; 2, *A. pacifica* ex *Beringraja binoculata*, Puget Sound; 3, *A. pacifica* ex *Raja rhina*, Puget Sound, USA; 4, *A. pacifica* ex *Raja stellulata*, Puget Sound, USA; 5, *A. pacifica* ex *B. binoculata*, Friday Harbour, USA; 6, *A. pacifica* ex *R. rhina*, Friday Harbour, USA; 7, *A. pugetensis* ex *B. binoculata*, Friday Harbour, USA; 8, *A*. *pugetensis* ex *B. binoculata*, off San Francisco USA; 9, *A. verrilli* ex unidentified skate, Cape Cod, USA; 10, *A. verrilli* ex *Leucoraja erinacea*, off Maine, USA; 11, *A. verrilli* ex *Amblyraja radiata*, off Newfoundland, Canada; 12, *A. verrilli* ex *A. radiata*, continental slope between northern Norway and Spitsbergen; 13, *A. grenii* ex *Raja clavata*, off Plymouth, UK; 14, *A. lobianchi* ex *R. clavata*, off Naples, Italy; 15, *A. lobianchi* ex *Bathyraja brachyurops*, off Plymouth, UK; 16, *A. lobianchi* ex *Raja microocellata*, off Plymouth, UK; 17, *A. lobianchi* ex *R. clavata*, off Plymouth, UK; 18, *A. lobianchi* ex *Raja montagui*, off Plymouth, UK; 19. *A. lobianchi* ex *Leucoraja naevus*, off Plymouth, UK; 20, *A. elegans* ex *R. clavata*, off Plymouth, UK; 21, *A. elegans* ex *R. clavata*, off Naples, Italy; 22, *Acanthocotyle* sp. ex *R. clavata*, off Plymouth, UK; 23, *Acanthocotyle* sp. ex *Narcine maculata*, off Plymouth, UK; 24, *Acanthocotyle* sp. ex *R. microocellata* Atlantic continental shelf off the mouth of the estuary Muros e Noia, Spain; 25, *Acanthocotyle* sp. ex *B. brachyurops* Atlantic continental shelf off the mouth of the estuary Muros e Noia, Spain; 26 *A. imo* n. sp. ex *Amblyraja frerischi*, off Tocopilla, Chile; 27, *A. atacamensis* n. sp. ex *Bathyraja peruana*, off Tocopilla, Chile; 28, *A. gurgesiella* ex *Gurgesiella furvescens* off Valparaiso, Chile; 29, *A. patagonica* ex *B. brachyurops*, Patagonian Shelf, Falkland Islands, UK; 30, *Acanthocotyle* sp. ex *Sympterygia bonapartii*, off Puerto Deseado, Argentina; 31, *A. urolophi* ex *Urolophus cruciatus*, off Tasmania, Australia

The analysis of the geographical distribution of members of *Acanthocotyle* suggests a close association with the temperate region (Fig. 7), although this conclusion should be considered with caution. To date, all known species have been described from fishes of two families of the Rajiformes (Rajidae and Arhynchobatidae), except for *A. urolophi* and *Acanthocotyle* sp. (Myliobatiformes and Torpediniformes, respectively). Regarding hosts of the Rajiformes, a search in the ISI Web of Sciences (1975–2018) and Scopus (1990–2018), using as search criteria “Rajidae”, “Rajiformes” and “parasites”, yielded 46 references (excluding records from freshwater Rajiformes) that include parasitological records for just 61 host species. The known species count of Rajiformes is 287 [36], and the geographical range of distribution of members of this order includes from tropical to polar seas, and from shallow to deep-waters in the Atlantic, Indian and Pacific Ocean [32] and only 14 species of Rajiformes have been recorded as host for members of *Acanthocotyle*. As stated in a previous study [40], Rajidae have particularly been neglected in terms of limited sampling effort when studying cestodes. This limitation also applies to other parasites, such as the monogeneans, and therefore clarifying patterns of distribution will require a substantial increase in research effort, particularly for deep-sea hosts. The current distribution of members of *Acanthocotyle* (Fig. 7) can thus be explained by intensive sampling effort in some localities, specifically off the Pacific and Atlantic coasts of North America and the English Channel. It is early to consider host specificity of *Acanthocotyle* even more if *c.*104 species of Rajidae are considered as deep-sea skates [41], and only four species (including this record) have been studied as host for species in *Anthocotyle*.

## Conclusions

Two new species of the genus *Acanthocotyle* are described from the skin of two deep-sea skates (Rajiformes) obtained at a depth of *c.*1500 m off Tocopilla (northern Chile). Both species represent the deepest record for members of *Acanthocotyle*. Conclusions about host specificity as well as geographical distribution of *Acanthocotyle* should be treated with caution due to the low proportion of Rajiformes studied for monogeneans. *Acanthocotyle* spp. have been recorded for 14 of the 287 species in Rajiformes. Future studies regarding parasites of Rajiformes are needed in order to evaluate the real level of host specificity and geographical distribution of members of *Acanthocotyle.*

## Supplementary information

**Additional file 1: Table S1.** *Acanthocotyle imo* n. sp. ex *Amblyraja frerichsi* (Krefft) (Rajiformes: Rajidae). Raw morphometric and meristic data.

**Additional file 2: Table S2.** Summary of morphological and morphometric characteristics of the species of *Acanthocotyle* considered valid.

**Additional file 3: Table S3.** *Acanthocotyle atacamensis* n. sp. ex *Bathyraja peruana* McEachran & Miyake, 1984 (Rajiformes: Arhynchobatidae). Raw morphometric and meristic data.

**Additional file 4: Table S4.** Code for recorded species of *Acanthocotyle* as shown in Fig. 7. Host species, family and order plus geographical record and authority are also given.

## Data Availability

Data supporting the conclusions of this article are included within the article. The sequences generated in this study were deposited in the GenBank database under the accession numbers MH511079–MH511082 (*A. atacamensis* n. sp.), MH511083-MH511085 (*A. imo* n. sp.) (*LSU* rRNA gene) and MH511067-MH511072 (*A. atacamensis* n. sp.), MH511074–MH511078 (*A. imo* n. sp.) (*cox*1 gene). Raw measurements of all specimens studied are included in Additional file 1: Table S1 and Additional file 3: Table S3. Code for recorded species of *Acanthocotyle* (as shown in Fig. 6) and host species, family and order plus geographical record and authority are given in Additional file 4: Table S4.

## Abbreviations

SPO: South-East Pacific
*LSU* rRNA: large subunit of rRNA
*cox*1: cytochrome *c* oxidase subunit 1 gene
AFA: alcohol: formalin: acetic acid
PCA: principal components analysis
TL: total length
MDA: multivariate discriminant analysis
PCR: polymerase chain reaction
BI: Bayesian inference
ML: maximum-likelihood
AIC: Akaike information criterion
GTR + G: General Time Reversible model + Gamma distribution
GTR + I: General Time Reversible model + Invariant sites
MCMC: Markov chain Monte Carlo
ESS: Effective sample size
